# Snow Leopard and Himalayan Wolf: Food Habits and Prey Selection in the Central Himalayas, Nepal

**DOI:** 10.1371/journal.pone.0170549

**Published:** 2017-02-08

**Authors:** Madhu Chetri, Morten Odden, Per Wegge

**Affiliations:** 1 Faculty of Applied Ecology and Agricultural Sciences, Inland Norway University of Applied Sciences, Campus Evenstad, Norway; 2 National Trust for Nature Conservation, Khumaltar, Lalitpur, Nepal; 3 Department of Ecology and Natural Resource Management, Norwegian University of Life Sciences, Ås, Norway; Université de Sherbrooke, CANADA

## Abstract

Top carnivores play an important role in maintaining energy flow and functioning of the ecosystem, and a clear understanding of their diets and foraging strategies is essential for developing effective conservation strategies. In this paper, we compared diets and prey selection of snow leopards and wolves based on analyses of genotyped scats (snow leopards n = 182, wolves n = 57), collected within 26 sampling grid cells (5×5 km) that were distributed across a vast landscape of ca 5000 km^2^ in the Central Himalayas, Nepal. Within the grid cells, we sampled prey abundances using the double observer method. We found that interspecific differences in diet composition and prey selection reflected their respective habitat preferences, i.e. snow leopards significantly preferred cliff-dwelling wild ungulates (mainly bharal, 57% of identified material in scat samples), whereas wolves preferred typically plain-dwellers (Tibetan gazelle, kiang and argali, 31%). Livestock was consumed less frequently than their proportional availability by both predators (snow leopard = 27%; wolf = 24%), but significant avoidance was only detected among snow leopards. Among livestock species, snow leopards significantly preferred horses and goats, avoided yaks, and used sheep as available. We identified factors influencing diet composition using Generalized Linear Mixed Models. Wolves showed seasonal differences in the occurrence of small mammals/birds, probably due to the winter hibernation of an important prey, marmots. For snow leopard, occurrence of both wild ungulates and livestock in scats depended on sex and latitude. Wild ungulates occurrence increased while livestock decreased from south to north, probably due to a latitudinal gradient in prey availability. Livestock occurred more frequently in scats from male snow leopards (males: 47%, females: 21%), and wild ungulates more frequently in scats from females (males: 48%, females: 70%). The sexual difference agrees with previous telemetry studies on snow leopards and other large carnivores, and may reflect a high-risk high-gain strategy among males.

## Introduction

Top carnivores play an important role in maintaining energy flow and functioning of ecosystems. They traverse large areas to fulfill their energy demands, and their wide ranging movements and killing of domestic stock create conflicts with pastoral communities [1]. Hence, a clear understanding of diets and foraging strategies of top predators is essential for developing effective conservation strategies. The snow leopard (*Panthera uncia*) is categorized as Endangered on the IUCN Red List, and it is listed in Appendix I of CITES. Retaliatory killing, poaching for wildlife trade, habitat degradation and prey depletion are considered key factors leading to population decline [2]. In contrast, wolves are listed in the category Least Concern. However, in the Himalayas, wolves are very rare, and a recent genetic study confirmed that they belong to the ancient Himalayan wolf lineage (*Canis lupus chanco*) [3]. Ecological information about wolves in this landscape is practically non-existent. However, the preferred habitat of wolves in mountain ranges, i.e. open grassland and alpine meadows [4], is frequently used by pastoral herding communities, and wolves are potentially vulnerable to retaliations due to livestock depredation.

Little is known about the nature of the coexistence of snow leopards and wolves, but recent studies in Kyrgyzstan and northwestern China revealed pronounced interspecific diet overlap [4, 5], and therefore a potential for exploitative competition. However, interspecific competition probably varies among regions depending on several interacting factors, such as prey abundance, prey species diversity and habitat heterogeneity, as these are known to promote interspecific coexistence through niche segregation [6–8]. In general, snow leopards prefer steep terrain, ridges, broken cliffs and gullies associated with alpine and sub-alpine pastures [9], whereas wolves prefer open undulating pastures associated with alpine meadows [10]. Hence, given that preferred habitats are available to both species, coexistence may be enhanced by spatial avoidance and the utilization of different prey species that are associated with these habitats.

A recent review and meta-analysis of prey preferences of snow leopards revealed a relatively narrow dietary niche breadth despite marked diet differences among regions [11]. A general trend was a preference for prey species within the size range of 36–76 kg, and if available, Siberian ibex (*Capra sibirica*), bharal (or blue sheep/naur *Pseudois nayaur*) and Himalayan tahr (*Hemitragus jemlahicus*) were identified as key prey species [11]. In the absence of preferred larger prey, snow leopards consume a larger proportion of various smaller species of sub-optimal size [11]. The wolf, on the other hand, appears to be a more generalist forager than the snow leopard [12]. Although information on wolf diets are completely lacking from the Central Himalayas, food habits have been studied extensively in North America and Europe [13]. The studies reveal that wolves are opportunistic predators, with diets varying widely among regions due to different prey availability. Hence, wolves exploit a wide range of prey species from large to medium-sized wild and domestic ungulates, to berries and fruits and even garbage [13]. However, although wolves exhibit a wide food niche, their degree of diet specialization seem to vary among regions [14]. In a manner similar to snow leopards, wolves tend to widen their foraging niche in areas where preferred larger ungulates are few [15].

In our study, we assessed factors associated with variation in diets and selection of prey among snow leopards and wolves on a large spatial scale (ca 5000 km^2^) to reveal the nature of their coexistence and their dependence on wild and domestic prey. Based on analyses of scats and the distribution and abundances of prey, we investigated four predictions regarding spatio-temporal patterns of diet composition. We predicted that the interspecific differences in habitat selection between wolves and snow leopards should be reflected in their diets and prey selection (i), wolves select typical plain-dwelling ungulates e.g. livestock, Tibetan argali (*Ovis ammon hogdsoni)*,kiang (*Equus kiang*), whereas snow leopards select typical cliff-dwellers e.g. bharal and Himalayan tahr. Furthermore, we predicted that scats from areas with low wild ungulate densities contain larger proportions of small mammals/birds and livestock (ii), and diet contents should differ with respect to season of scat collection (iii) due to a changing prey availability caused by the winter hibernation of marmots, an important prey species in the Central Himalayas [11, 16]. Lastly, we expected diet contents to differ between sexes (iv), as males seem more prone to kill livestock, which is a pattern observed among GPS collared snow leopards [17] and several other carnivore species such as lynx [18] and common leopards [19].

## Study Area

**Fig 1:**
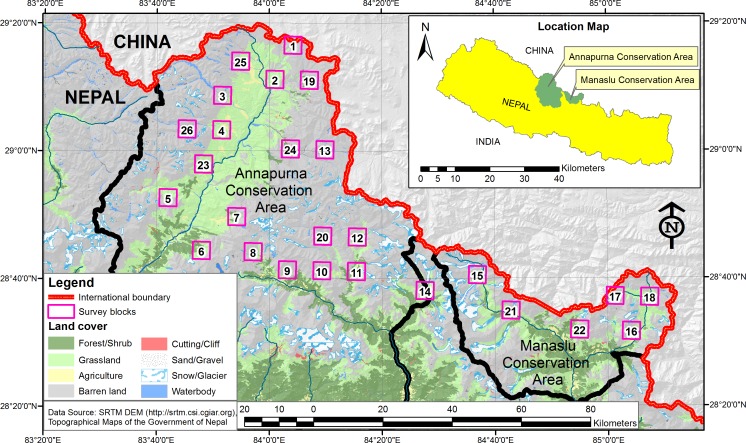
Study area: Location of survey grid cells in the Annapurna-Manaslu landscape.

The study area encompassed the Annapurna-Manaslu landscape (N28-29°, E83-85°, Fig 1), situated in the rain shadow of the Trans and semi-Trans Himalayas and adjoining the vast Tibetan Plateau in the north (Fig 1). A major proportion falls within the Annapurna Conservation Area (ACA) and the Manaslu Conservation Area (MCA), and a smaller proportion is in the Bhimthang valley which is situated between ACA and MCA.

For more than two decades, the National Trust for Nature Conservation (NTNC) has implemented a holistic conservation model for improving the rural livelihoods and conserving biodiversity in the two conservation areas. Climate in the region is highly variable with annual temperatures ranging from a minimum of -40°C in winter to a maximum of approximately 30°C in summer. The total annual rainfall is <200 mm and more than half of the total precipitation is snow during the winter months [20]. The northern part of the landscape is treeless and contains flat undulating plains dominated by graminoids (*Carex* spp. and *Kobresia* spp.) (Fig 1). The central part is dominated by cliffs and rugged terrain composed of limestone rocks, interspersed by open areas with scrub vegetation and rangelands. The area is unique in terms of both faunal and floral species whose distributions are mainly determined by vertical topography, altitudinal gradient and the aspect of slopes. The large mammalian fauna include bharal, Himalayan tahr, Tibetan argali, kiang, Tibetan gazelle (*Procapra picticaudata)*, Himalayan serow (*Capricornis thar*), and alpine musk deer (*Moschus chryogaster*). Predator species include snow leopard, Himalayan wolf, brown bear (*Ursus arctos*), Eurasian lynx (*Lynx lynx*), red fox (*Vulpes vulpes*), Tibetan fox (*Vulpes ferrilata*) and golden jackal (*Canis aureus*). Other smaller mammals are Himalayan marmot (*Marmota himalayensis)*, Wooly hare (*Lepus oiostolus)*, different species of small mustelids (*Mustela* spp., *Martes* spp.), small felids (*Felis* spp., *Prionailurus* spp.), and several species of pikas (*Ochotona* spp.) and voles (*Alticola* spp.).

Animal husbandry is the main subsistence economy of the local communities. Pastoral communities use all the available rangelands for their traditional rotational grazing practices and move to different pastures according to their traditional calendar [21]. The livestock assemblage includes goat (*Capra hircus*), sheep (*Ovis aries*), yak (*Bos grunniens*), cattle-yak hybrids (dzo, jhopas, *Bos* spp.), lulu cows (*Bos taurus* sp.) and horses (*Equus ferus caballus*). Although the available grazing land has been used by nomads for centuries, nomads are now less common, and most of the families are settled in villages [22].Sheep, goats and cows are usually herded and periodically moved among different pastures according to seasons. Milking yak cows are brought back to the corrals/pens in the afternoon or in the morning on a regular basis. Male yaks and horses are left free in the pasture and are visited at an interval of 1–3 months for salt feeding depending on the pasture types, season and accessibility. During summer livestock are taken to higher elevation for grazing, and in winter, they move to lower elevations due to the harsh weather conditions.

## Methods

### Ethics statement

Relevant permits required to carry out the research were obtained from the National Trust for Nature Conservation, Nepal.

### Sampling design

A random or systematic selection of sampling areas was not feasible due to logistic and cultural constraints. Hence, we placed a 5×5 km grid over a digitized map of the study area and used the following selection criteria: (i) Each of the 5×5 km sampling grid cells were situated with a minimum distance of 5 km and a maximum of 10 km to the border of the nearest grid cell, (ii) we avoided using grid cells with more than 50% of glaciers and inaccessible high mountain peaks, (iii) we avoided areas falling in and around larger settlements and areas with other cultural restrictions (e.g. only allowed to be visited in certain seasons by local communities), (iv) we avoided areas along the main trekking routes and the main road (Korolla-Jomsom highway in ACA), and (v) we avoided areas below 3000 m a.s.l., as snow leopard presence had not been reported in these low altitudes in this region [9]. A preliminary survey was conducted during April-June 2012 to validate the feasibility of the identified sampling cells before the beginning of the data collection. All the selected cells were located within the distribution range of snow leopards [23, 24]. Only one of the grid cell was outside the conservation areas, in the Bhimthang valley. Altogether, the grid cells covered 650 km^2^, 13% of the total study landscape.

### Scat collection

We collected scats during the warmer (May-October) and the colder (November-April) seasons in each of the 26 grid cells (Fig 1). Putative snow leopard (N = 573) and wolf scats (N = 236) were collected along trails, mountain ridges, river beds and mountain passes [25]. Altogether, 567 km were covered during 151 days of field work, rendering an average of 22 km in each grid cell (min = 5.83, max = 39.92, SD = 9.06). Once a scat was encountered, a small part was placed in a plastic tube with silica desiccant [26] for DNA fecal analysis. Larger parts were stored in paper envelopes for diet studies. We left ca half of the scat in the field to avoid disturbing the regular movements and territorial marking of the predators [27].

### Scat analyses

We screened the samples prior to genotyping. First, we disregarded scats that seemed too old to contain DNA material, i.e. scats that were cracked, with a white or light yellowish color, had no odor, or were infected by fungus. Secondly, due to economic limitations, we set a maximum upper limit of 30 scats/grid cell. Scats were genotyped to identify species and sex following the methods described by Karmacharya et al.[28] and Kocher et al.[29]. Among a total of 347 and 100 snow leopard- and wolf scats analyzed, only 182 and 57 were successfully verified. The majority of the other samples had too low DNA quality, and some wolf scats were identified as feral dogs (1 scat), lynx (1 scat) and brown bear (1 scat).

Reference hair samples of potential prey were collected from the remains of kills found in the study area, and we collected hair of livestock from the villages nearby. Guard hair from different parts of the body were put in paper envelopes and annotated systematically. We prepared a photographic identification key of 16 wild and domestic animals following the methods described in Oli et al.[16] and Bahuguna et al.[30]. The genotyped scat samples were first oven-dried for 24 hours at 90°C and then washed using detergents and sieved under running water. We placed two sieves on top of each other to extract hairs, one with a mesh size of 0.8 mm for larger/regular hairs and 0.5 mm below for small mammal hairs [4]. Substances such as bones, claws, bird feathers, stones, and plant material were manually separated, and then dried and stored in plastic bags. In order to prepare hairs for species identification, we followed the same procedure as described above. To select hairs, we used a modified version of the point frame method [31–33]. Using a gridded tray, we selected 50 different hairs closest to grid intersections and compared them with the prepared reference slides based on cuticular cell arrangements, medullary patterns and relative lengths [30, 34]. Small rodents and birds were not identified to species, but grouped in two different categories. For snow leopard and wolf scats, we were unable to identify 3.6% and 1.9% of the analyzed material, respectively.

### Prey abundance

Among the different methods of abundance estimation of ungulate populations, distance sampling is a widely used method in tropical and temperate forests [35], but in the Himalayas, this method is not feasible due to the ruggedness of the landscape [36–38]. Instead, we used the double-observer survey method, as this technique has been evaluated as statistically robust and sufficiently precise for estimates of mountain ungulates [38, 39]. Within each grid cell, we followed trails made by humans and livestock and stopped at vantage points for scanning the area for 15–20 minutes. Two groups counted animals along the same trail with a time interval of 45–60 minutes. All wild and domestic ungulates, and smaller mammals (marmots and wooly hares), were recorded using binoculars and spotting scopes. For each observed group of prey, we noted the GPS location, the time of day, age and sex (if possible), vegetation, aspect, slope and altitude. After each survey, the two groups met to compare the data to confirm the unique identity of each sighted group [39]. Within each grid cell, we systematically mapped the actual areas covered by the survey (areas seen) by using 1:25.000 topographic paper maps, and the areas that were inaccessible from the trails and vantage points (areas not seen) were subtracted. This was done to avoid overestimating the size of the area surveyed, and thereby avoiding underestimation of animal densities.

### Statistical analyses

We used the program R (version 3.2.5) for all statistical analyses, except for the analyses of prey selection where we used the SCATMAN software [40].

#### Prediction 1

For direct comparisons of scat contents between snow leopards and wolves, we only used samples that had been collected in six grid cells in the northwestern part of the study area where genotyped scats from both predators have been found (snow leopard: n = 27; wolf: n = 47). This was done to ensure that observed differences was due to selection of different prey species rather than differences in prey availability. We compared the proportions of four food categories between the two species using Fisher’s exact tests, i.e. cliff- dwelling wild ungulates (bharal and Himalayan tahr), plain-dwelling wild ungulates (Tibetan argali, Tibetan gazelle and kiang), livestock and small mammals/birds. Before the tests were executed, we converted the relative proportions of recognized prey material into “Whole Scat Equivalents” (WSE) by multiplying the proportional occurrence of each food category (the proportions relative to all analyzed material) in all scats with the total number of scats [41]. This method allows for comparisons of proportions of food items in scats without altering the total sample size. For instance, if 100 scats all contained 10% of small mammals; they would convert into 10 scats of small mammals and 90 scats of other material. In the tests, we compared the WSEs of each food category vs. other material. We used Pianka’s index [42] to assess diet overlap (DO) between the two predators; DO = $\frac{\sum PijPik}{\sqrt{\sum P^{2}ij+\sum P^{2}ik}}$ where Pij is the proportion of prey category i in the diet of predator j; Pik is the proportion of prey category i in the diet of predator k. The values range between 0 (no overlap) and 1 (complete overlap).

We analyzed prey selection based on the whole scat material (snow leopard: n = 182; wolf: n = 57) comparing observed and expected relative proportions of food items in scats with likelihood ratio tests (G-tests) [43], and a parametric bootstrapping procedure to obtain the p-values [44]. We specified the proportional occurrences in scats of three food categories; cliff- and plain-dwelling wild ungulates and livestock, and their estimated densities with standard errors. We also included estimates of scat production rates for each food category by using conversion equations for snow leopards (Y = 1.980 + 0.035X, where Y = the biomass of prey consumed to produce a scat, and x = the average body weight of each prey species) [45] and for wolves (Y = 0.439 + 0.008X) [46]. This was done to take into account that the relative amount of scats produced per ingested volume of different prey items varies with the size of the prey. Smaller prey produces more scat material per weight unit than larger prey due to a larger ratio of surface/volume [45]. As indices of prey selectivity, we used the Jacobs index [47] i.e. D_i_ = (r_i_—a_i_)/(r_i_+a_i_-2r_i_a_i_), where r_i_ = proportional occurrence of prey items in the scats, a_i_ = proportional availability of a given prey item relative to all available prey. The value of Di ranges from +1 (maximum preference) to—1 (maximum avoidance).

#### Predictions 2–4

We assessed factors influencing diet composition of snow leopards and wolves with Generalized Linear Mixed Models (GLMM). As binomial response variables, we used the presence or absence of three main food categories; wild ungulates, livestock and small mammals/birds. We used sampling grid cell ID as a random factor in all models. We used the following explanatory variables: The sex of the predator, the season of scat collection, and characteristics of the sampling grid cells including latitude, longitude, density of wild ungulates, density of livestock and wild prey species diversity (Shannon-Wiener index: H' = -$\sum_{i=1}^{s}pi\ln pi$, where pi = proportion of individuals of i-th species, s = number of species). We started by investigating models with the terms sex, season, sex + season, and the NULL model with only the intercept and the random factor, by comparing their Akaike Information Criterion values [48]. We selected the most parsimonious model with the lowest AIC value and added the different grid cell specific variables listed above, one at the time. For wolf scats we only investigated the influence of season, as samples were found in only six grid cells and the number of scats with known sex was small. We also used the AICc instead of AIC due to a small sample size.

Throughout the text, scat contents are presented either as percentages (%) of each identified food item relative to all recognized material, or as frequencies of occurrence (FO). The latter is the percentage (%) of scats that contain a given food item.

## Results

### Prey density and distribution

The most commonly encountered cliff-dwelling wild ungulate was the bharal, which occurred in high densities in the majority of the study grid cells (Table 1). The other cliff-dwelling ungulate, the Himalayan tahr, was only found in four grid cells in the eastern part (MCA). Tibetan argali, Tibetan gazelle and the kiang were found only in two grid cells in the northwestern part (Upper Mustang of ACA), and their densities were low (Table 1). Domestic animals were generally more evenly distributed (except the lulu cow, Table 1) than the wild ungulates. The highest average density was recorded for goats (Table 1), followed by yak, sheep, horse and the lulu cow. The overall density of domestic livestock (35.74 ± 0.10/km^2^) was far higher than that of the wild ungulates (7.41 ± 0.09/km^2^). We did not attempt to estimate densities of smaller mammals. However, the most commonly observed species, Himalayan marmots, wooly hare and Royle’s pika were present in 69.2%, 65.4% and 100% of the grid cells, respectively.

**Table 1:** Average densities of wild and domestic ungulates in 26 study grid cells (5×5 km each) in the Central Himalayas.

Species	Density (No/km^2^±SE)	Biomass (kg/km^2^)	Species presence in grid cells (%)
**Wild ungulates**			
Bharal	5.97 ± 0.10	202.98	88.46
Himalayan Tahr	0.85 ± 0.22	42.50	15.38
Tibetan argali	0.24± 0.40	19.20	7.69
Tibetan gazelle	0.09 ± 0.14	1.80	7.69
Kiang	0.25 ± 0.12	25.00	7.69
**Livestock**			
Goat	16.39 ± 0.16	409.75	65.38
Sheep	6.36 ± 0.09	190.80	65.38
Horse	1.24 ± 0.06	136.40	73.08
Yak/Chauri	10.92 ± 0.14	1638.00	92.31
Lulu Cow	0.83 ± 0.04	83.00	42.31

### Diet comparison between snow leopards and wolves

Overall, the scats of snow leopards consisted of 73% prey of wild origin and 27% of domestic animals. Among the different categories of wild prey, cliff- dwelling ungulates (bharal and Himalayan tahr) dominated the diet (57% of remains in scats), and the most commonly identified species was the bharal (Table 2). Plain-dwelling ungulates (kiang, Tibetan argali and Tibetan gazelle) were almost absent in the scats (1%), whereas small mammals constituted 13%. Among domestic animals, the highest proportion in the scats was from goats, followed by horses, sheep, yak and lulu cow (Table 2). Altogether, five scats contained twigs of tamarisk *Myricaria* spp..

**Table 2:** Diets of snow leopard and wolf: proportions (%) of wild and domestic prey in scats, and estimated proportions of biomass and individuals consumed.

Species	Snow leopard (N = 182)			Himalayan wolf (N = 57)		
	Proportions in scats (%)	Relative biomass consumed (%)	Relative number of prey individuals consumed (%)	Proportions in scats (%)	Relative biomass consumed (%)	Relative number of prey individuals consumed (%)
**Wild prey**	** **		** **		** **	
***Cliff***	** **		** **		** **	
Bharal	56.85	56.16	18.32	4.42	4.09	1.76
Himalayan tahr	0.55	0.64	0.14	-	-	-
***Plain***						
Tibetan argali	0.78	1.16	0.16	8.95	12.57	2.30
Tibetan gazelle	0	0.00	0.00	11.05	8.62	6.31
Kiang	0	0.00	0.00	10.84	17.48	2.56
***Small***						
Himalayan marmot	6.55	4.47	8.26	32.11	20.36	49.66
Wooly hare	3.52	2.33	6.45	4.81	2.95	10.79
Royle's pika	2.53	1.57	58.02	0.67	0.38	18.78
*Unidentified rodents*	0.89	-	-	3.05	-	-
*Unidentified birds*	1.09	-	-	0.32	-	-
**Livestock**	** **	** **				
Yak	2.2	4.96	0.37	4.18	8.92	0.87
Horse	6.31	11.46	1.16	5.26	9.03	1.20
Lulu cow	0.41	0.70	0.08	4.6	7.42	1.09
Goat	13.99	12.45	5.52	8.53	7.10	4.15
Sheep	4.35	4.11	1.52	1.23	1.09	0.53

Small mammals was the most common food category found in wolf scats (41%) followed by plain dwelling ungulates (31%). Only a small proportion of the scat material (4%) was from cliff dwelling ungulates. Among domestic animals (24%), the highest proportion in the wolf scats was from goats, followed by horses, lulu cow, yak and sheep (Table 2). Three wolf scats contained plant material (*Kobresia* sp. and *Pennisetum* sp.), and two scats contained plastic material.

Fisher exact tests revealed significantly different proportions of cliff-dwelling (P = 0.001) and plain-dwelling wild ungulates (P = 0.001) between snow leopards and wolves (Table 3). The proportion of domestic animals was significantly higher in scats of snow leopards than among wolf scats (P = 0.025), whereas small mammals tended to be higher in wolf scats, but not significantly (P = 0.081). The Pianka index value was 0.44, thus indicating a relatively small diet overlap between the two species.

**Table 3:** Selection of ungulate prey among snow leopards and wolves in the Central Himalayas.

Species	Category	Obs	Exp	χ2	P-value	Jacob's index
Snow leopards (n = 49)	Cliff	104	20	418.3	0.000	0.9
	Plain	1	3	1.7	0.196	-0.6
	Livestock	49	131	344.8	0.000	-0.8
Wolves (n = 14)	Cliff	3	15	18.4	0.000	-0.8
	Plain	17	1	171.5	0.000	0.9
	Livestock	14	17	1.1	0.316	-0.2

### Prey selection

Patterns of selection among the three categories of ungulates, i.e. cliff dwellers, plain dwellers and livestock, differed markedly between snow leopards and wolves. Snow leopards exhibited a significant preference for cliff-dwelling wild ungulates and a significant avoidance of livestock (Table 3). The selection index for plain dwelling ungulates was negative, thus indicating avoidance, but the difference in between use and availability was not significant (Table 3). The wolves exhibited a significant preference for plain dwelling ungulates and an avoidance of cliff dwellers, whereas livestock was consumed according to their availability (Table 3). The sample size of wolf scats was too small to test for selection among different livestock species. However, snow leopards significantly preferred horses and goats, and avoided yaks (Table 4). No selection was observed for sheep and lulu cows.

**Table 4:** Selection of livestock by snow leopards in the Central Himalayas.

Species	Livestock	Obs	Exp	χ2	P-value	Jacob's index
Snow leopards (n = 50)	Horse	12	3	35.1	0.000	0.7
	Goat	25	17	5.4	0.032	0.3
	Sheep	8	7	0.0	0.836	0.0
	Yak	4	21	24.0	0.000	-0.8
	Lulu	1	2	0.2	0.630	-0.2

### Factors influencing diet composition

For the occurrence of main food categories in the snow leopard scats, we tested for the relative influence of the sex of the predator, the season of scat collection, the latitude and longitude of the scat locations, the densities of wild ungulates and livestock, and wild prey species diversity (Table 5, data in S1 Appendix). Among the models for wild ungulate remains, two out of 14 candidate models had ΔAIC values <2, thus indicating small differences in performance between them. However, both models included the term “sex”, and the highest ranking model (M5, Table 5) also included latitude (Y-coordinate), whereas the second best model (M4) included longitude (X-coordinate). Wild ungulates occurred in a larger proportion of samples from females (70%) than those from males (48%). The parameter estimates from the best model of wild ungulate occurrence (M4, Table 6) were negative for longitude and positive for latitude. This means that the occurrence of wild ungulates increased from south towards north and decreased from west towards east.

**Table 5:** Generalized linear mixed models of factors influencing diet composition of snow leopards in the Central Himalayas.

Model	Explanatory variables	Ungulates		Livestock	
		ΔAIC	W	ΔAIC	W
M1	Sex+Season	3.7	0.05	5.5	0.04
M2	Sex	3.2	0.07	3.5	0.10
M3	Season	10.2	0.00	15.0	0.00
M4	Sex+X	0.2	0.31	5.3	0.04
M5	**Sex+Y**	**0.0**	**0.34**	**0.0**	**0.61**
M6	Sex+ DD	4.8	0.03	3.6	0.10
M7	Sex + DU	4.8	0.03	5.3	0.04
M8	Sex + SW	2.3	0.11	5.4	0.04
M9	X	6.2	0.01	15.0	0.00
M10	Y	4.7	0.03	8.4	0.01
M11	DD	10.4	0.00	12.4	0.00
M12	DU	10.7	0.00	15.0	0.00
M13	SW	8.7	0.00	15.2	0.00
M14	NULL	9.1	0.00	13.3	0.00

The binomial response variables were the presence of wild ungulates and livestock in scats. X and Y indicates longitude and latitude coordinates (standardized UTM X and UTM Y values). DD = density of livestock; DU = density of wild ungulates; SW = wild prey species diversity expressed as the Shannon-Wiener Diversity Index. ΔAIC = the difference in Akaike Information Criteria between each model and best model with the lowest AIC; W = Akaike weight.

**Table 6:** Parameter estimates and test statistics of Generalized Linear Mixed Models of diet composition of snow leopards and wolves in the Central Himalayas.

Species	Response variable	Predictor variable	Estimate	SE	Z-value	P
Snow leopards	Ungulate	Intercept	-1.96	0.62	-1.54	0.120
		Sex	0.90	0.35	2.57	0.010
		Latitude (Y)	0.59	0.29	2.02	0.040
	Livestock	Intercept	0.94	0.6	1.56	0.118
		Sex	-1.14	0.36	-3.20	0.001
		Latitude (Y)	-0.59	0.27	-2.22	0.026
	Small mammals	Intercept	-2.16	0.41	-5.30	0.000
		SW	0.94	0.36	2.27	0.024
Wolves	Ungulate	Intercept	3.19	1.18	2.70	0.000
		Seasons	-0.95	0.66	-2.94	0.000
	Small mammals	Intercept	-3.58	1.27	-2.82	0.000
		Seasons	2.30	0.72	3.20	0.000

Response variables were the occurrence of wild ungulates (Ungulate), livestock and small mammals/birds (Small) in scats. SW indicate wild prey species diversity expressed as the Shannon-Wiener Diversity Index.

The best model of livestock occurrence in snow leopard scats included the two predictor variables “sex” and “latitude” (M5, Table 5). The second best model (M2), with a ΔAIC value of 3.5 included only the term sex. The parameter estimate for latitude was negative, indicating that livestock occurred more frequently in scats collected in the southern part of the study area (Table 6). Furthermore, livestock occurred more than twice as frequently in scats from male snow leopards (47%) than in scats from females (21%). Regarding the food category “small mammals/birds”, the best model included only the wild prey species diversity index (M13, Tables 6 and 7). Samples containing this food category were found within grid cells with higher diversity indices (Tables 6 and 7). None of the other candidate models performed better than the NULL model, as their ΔAIC values were only marginally different.

**Table 7:** Generalized linear mixed models of factors influencing the occurrence of small mammals/birds in snow leopard scats in the Central Himalayas.

		Small mammals	
Model	Explanatory variables	ΔAIC	W
M1	Sex+Season	5.0	0.02
M2	Sex	4.3	0.03
M3	Season	3.3	0.05
M4	Seson+X	4.5	0.03
M5	Season+Y	5.3	0.02
M6	Season+ DD	3.6	0.04
M7	Season + DU	5.3	0.02
M8	Seson + SW	1.8	0.10
M9	X	2.9	0.06
M10	Y	4.5	0.03
M11	DD	2.7	0.07
M12	DU	4.5	0.03
M13	**SW**	**0.0**	**0.25**
M14	NULL	2.5	0.07

X and Y = longitude and latitude coordinates (standardized UTM X and UTM Y values). DD = density of livestock; DU = density of wild ungulates; SW = wild prey species diversity expressed as the Shannon-Wiener Diversity Index. ΔAIC = the difference in Akaike Information Criteria between each model and best model with the lowest AIC; W = Akaike weight.

The occurrence of domestic animals in wolf scats did not differ among seasons, as the null model had the lowest AICc-value (data in S2 Appendix). For wild ungulates, the AICc of the null model was 3.60 (Parameter estimate = -1.95±0.66SE), and for small mammals/birds it was 6.09 (Parameter estimate = 2.30±0.72SE). The models including the predictor variable “season” performed better in both cases (Table 6). Wild ungulates occurred far more frequently in scats from the cold season (78%) than from the warm season (33%). An opposite pattern was evident for small mammals/birds, as these occurred markedly more frequently in samples from the summer (74%) than from winter (22%).

## Discussion

Wolf scats contained larger proportions of plain dwelling ungulates and smaller portions of cliff dwellers than scats from snow leopards. Similarly, wolves significantly selected plain dwellers, whereas snow leopards selected cliff dwellers. Hence, prediction one was supported by our data. These predictions were founded on previously observed patterns of habitat selection among the two species, i.e. that wolves prefer open undulating plains associated with alpine meadows, whereas snow leopards are adapted to rugged terrain and cliffs [4, 9, 10]. Our results concur with these previous findings, as wolf scats were found mainly in the northwestern sampling grid cells where plain dwelling ungulates were most common. Apparently, the differences between the two species in diets and habitat use are associated with their hunting strategies and social behaviors. The hunting success of the snow leopard and other solitary and stalking/ambushing predators depends on access to cover to reduce attack distances [49]. Coursing and pack hunting predators are typically less dependent on cover, and their hunting success have been attributed to several factors such as pack sizes and physical characteristics of the prey [50, 51].

In our interspecific diet comparison, we found that snow leopard scats contained a larger proportion of livestock than wolf scats. Nevertheless, the prey selection analyses revealed significant avoidance of livestock among snow leopards, but not among wolves. These seemingly contrasting results are probably caused by the interspecific comparison being based on samples from areas where both species were present, whereas prey selection was analyzed based on the whole scat material. Both species were present in the northwestern part which contained relatively large areas of alpine meadows and less rugged terrain. Hence, the prime prey of snow leopards in our study area, the bharal, was few, and this may have caused a relatively high utilization of livestock among snow leopards compared to wolves. Regarding selection among livestock species, snow leopards preferred horses, but avoided yaks. A higher attack frequency on horses has also been observed in previous studies [52], and it may be caused by horses often being left unattended in the pastures. Yaks are also unattended, but they are generally too large to be killed by snow leopards. Goats and sheep are prey of optimal size for snow leopards, but only the former was significantly preferred. Both species are attended by herders, but goats are often more dispersed than sheep and therefore probably more vulnerable. Regarding interspecific diet overlap, the Pianka index value of 0.44 was much smaller than in two previous comparisons between the two species, i.e. 0.91 [4] and 0.87 [5]. Both previous studies attributed the high diet overlap to low prey diversity. Our results concur with this explanation, as species richness was relatively high in the northwestern part of our study area where the two carnivores overlapped in distribution.

We predicted that scats from areas with low wild ungulate densities should contain larger proportions of small mammals/birds and livestock, due to a lower degree of diet specialization towards preferred wild ungulate prey in these areas. However, this prediction was not supported, as the best models of small mammal and livestock occurrence in scats did not include wild prey abundance. For small mammals, the best model only included the term “wild prey diversity”, and the association was positive, but relatively weak. The Shannon-Weiner diversity index gives high values when the numbers of prey species are high and with relatively even densities. Hence, a low density of a dominating prey species in these areas, such as bharal, may have given high index values. As a consequence, the consumption of small mammals and birds may have been positively associated with the species diversity index due to a lower availability of the preferred bharal.

We observed seasonal diet differences among wolves, but not among snow leopards. Hence, prediction iii was only partially supported. Wolves consumed more small prey during summer and more wild ungulates during winter. Marmots was the most common small prey species in wolf scats, and the seasonal diet differences is probably caused by changes in the accessibility of marmots due to their winter hibernation. Although livestock are distributed in different altitudes during the warmer and colder months, we did not detect any seasonal difference in their occurrence in predator scats. Presumably, seasonal differences in the accessibility of livestock was not high enough to cause significant diet shifts.

Among snow leopards, scat contents differed between the sexes, i.e. females had a higher proportion of wild ungulates and males a higher proportion of livestock. Hence, prediction iv was supported and the results concur with a telemetry study of snow leopards in Mongolia, which showed that the proportion of livestock among located kills were more than twice as high among males than females [17]. More frequent livestock killing among males than females has also been observed among several other carnivore species [53], but to our knowledge, it has never before been revealed based on scat analyses. Male biased livestock killing in carnivores has been suggested to be caused by higher encounter rates among males due to their wider ranging movements [53]. It has also been suggested that sexual selection have favored a high risk-high gain strategy among the males [19, 54], which involves the depredation of easily accessible domestic prey even though it may impose a greater mortality risk due to retaliatory killing.

Livestock depredation and the associated conflicts with humans is a main challenge for the conservation of both snow leopards and wolves [15, 55]. In this context, an interesting aspect is whether management aimed at enhancing the wild prey base will lead to reduced predation rates on domestic animals [56]. In a study of Eurasian lynx, livestock depredation was inversely related to wild ungulate prey density [18]. Furthermore, a recent review revealed a somewhat similar relationship among larger felids [57], i.e. that the proportion of livestock in diets abruptly increased when wild prey biomass decreased below a threshold value. In our study, we did not detect any influence of wild prey density on the occurrence of livestock in snow leopard scats even though wild prey biomass within sampling grid cells ranged both above and below the suggested threshold value of 545 kg/km^2^ [57]. The lack of a relationship in our study may be partially due to a confounding effect of the wide ranging movements of snow leopards. Recently, snow leopards in Mongolia was shown to move within home ranges of several hundred km^2^ [58], and this entails that some individuals in our study area may have utilized several different sampling grid cells. Although thresholds in livestock depredation may exist, we believe that more research is needed to the potentially interacting effects of livestock- and wild prey abundance on the probability of livestock killings.

## Supporting Information

S1 Appendix Snow leopard diet data for logistic models (Snowleopardsdiet.txt). (TXT)

S2 Appendix Wolf diet data for logistic models (Wolfdiet.txt). (TXT)
